# TGF-β signalling drives chemotaxis of human induced pluripotent stem cell-derived cardiomyocytes in response to MI stimulus

**DOI:** 10.1007/s00395-026-01201-9

**Published:** 2026-08-12

**Authors:** Laura Deelen, Kazuya Kobayashi, Gowtham Reddy Cheruku, Alia Hafiz Abbas Gasim, Fiona Lewis-McDougall, Ken Suzuki

**Affiliations:** Centre for Microvascular Research, Faculty of Medicine and Dentistry, William Harvey Research Institute, Queen Mary University of London, London, UK

**Keywords:** Human induced pluripotent stem cell-derived cardiomyocytes (hiPSC-CMs), Myocardial infarction (MI), TGF-β signalling, Chemotaxis, Cardiac repair

## Abstract

Human induced pluripotent stem cell-derived cardiomyocytes (hiPSC-CMs) hold significant promise for cardiac regeneration therapies. However, the efficacy of such treatments depends on the ability of transplanted cells to migrate and integrate into the damaged myocardium, a process that remains poorly understood. In this study, we investigated the migratory behaviour of hiPSC-CMs using homogenised rat MI tissue to simulate myocardial infarction (MI) in vitro. Transwell migration assays demonstrated a concentration-dependent chemotactic response, with hiPSC-CM migration increasing up to threefold towards MI tissue homogenate. Wound-healing assays further confirmed enhanced migration under MI-mimetic conditions. Bulk RNA sequencing revealed activation of the TGF-β signalling pathway as a key regulator of this response. Inhibition of TGF-β signalling, both pharmacologically and through antibody neutralisation, significantly reduced hiPSC-CM migration. These findings uncover a previously underappreciated chemotactic capability of hiPSC-CMs and identify TGF-β signalling as a central mediator, offering new mechanistic insights and potential therapeutic targets to improve the integration and efficacy of hiPSC-CM-based cardiac regeneration strategies.

**Supplementary Information:**

The online version contains supplementary material available at https://doi.org/10.1007/s00395-026-01201-9.

## Introduction

Myocardial infarction (MI) is one of the most common causes of heart failure (HF), with an estimated 5 year survival rate of approximately 50% following HF diagnosis [39]. During MI, persistent coronary artery obstruction leads to the irreversible loss of cardiac cells including cardiomyocytes (CMs) [14]. Currently, the only established treatment for end-stage HF patients is heart transplantation or the permanent implantation of a left-ventricular assist device as an emerging alternative [21, 37]. However, these approaches have several limitations, including high costs, complications related to immunosuppression or anticoagulants, and psychological strain for patients. Myocardial regeneration therapy has drawn major attention over the last few decades as an innovative treatment to restore functional CMs after MI and improve prognosis through enhanced cardiac function [8]. Human induced pluripotent stem cells (hiPSCs) have gained popularity as donor cells for myocardial regeneration therapy due to unlimited self-renewal, autologous clinical potential, and robust cardiomyogenic differentiation potency. Therefore, the transplantation of hiPSC-derived CMs (hiPSC-CMs) has become an active field of research for the treatment of post-MI HF.

Promising methods for delivery of replacement CMs to the heart include epicardial placement and intramyocardial injection of cell suspension or spheroids [19, 31, 36]. The migration of hiPSC-CMs following transplantation is a critical determinant of the success of cell-based cardiac regenerative therapies. Following epicardial delivery, a significant proportion of hiPSC-CMs have been shown to remain localised on the heart surface, failing to penetrate and integrate with the host myocardium [13, 29]. Whilst intramyocardial injection offers more precise delivery into damaged myocardial regions, even this method relies on the transplanted cells’ ability to migrate within the myocardium to establish functional integration with surviving host CMs. Such migration is essential for structural continuity, electrical coupling, and functional contribution to the regenerating heart. Therefore, limited intramyocardial migration remains a major barrier to the success of cell-based therapies.

Previous studies have begun to shed light on the migratory capacity of human stem cell-derived CMs. Moyes et al. demonstrated that human embryonic stem cell-derived CMs (hESC-CMs) exhibit enhanced migration in vitro in response to Wnt5a and fibronectin [30]. Another study identified BANCR, a primate-specific long non-coding RNA, as a positive regulator of hiPSC-CM migration [42]. Additional research on embryonic and neonatal CM migration has revealed that non-coding RNAs and developmentally regulated proteins may influence CM motility [2, 3, 15, 34].

Amongst the key pathways implicated in CM migration during regeneration is the TGF-β signalling pathway. In zebrafish, which possess innate cardiac regenerative capacity, SMAD3-dependent TGF-β signalling is essential for CM migration into damaged tissue and chemical inhibition of TGF-β signalling significantly impairs this migratory response [7]. Moreover, suppression of TGF-β–Smad3 signalling has been shown to inhibit an EMT-like response in CMs during zebrafish heart regeneration following ventricular ablation [33].

It is known that MI triggers the release of numerous signalling molecules into the surrounding tissue, some of which may serve as chemoattractants for migratory cells. Whilst adult CMs likely lack the capacity to respond to these cues, hiPSC-CMs due to their foetal-like phenotype may retain an intrinsic ability to migrate in response to such signals, similar to regenerative responses observed in zebrafish [18].

In this study, we aimed to investigate the migratory behaviour of hiPSC-CMs in response to MI-associated signals. To this end, we employed Transwell migration and wound-healing assays to assess hiPSC-CM migration in vitro, using rat MI tissue homogenate to mimic the post-MI microenvironment. Furthermore, we conducted bulk RNA sequencing on migrated and non-migrated hiPSC-CMs to identify key molecular pathways involved in this response.

Together, this study provides novel mechanistic insights into the chemotactic and migratory behaviour of hiPSC-CMs in the context of MI. These findings provide new insights into the migratory behaviour of hiPSC-CMs in response to infarct-associated cues and highlight molecular targets, such as TGF-β signalling, that could be modulated to enhance cell integration and improve functional outcomes in post-MI cardiac regenerative therapies.

## Results

### HiPSC-CMs exhibit a concentration-dependent chemotactic response to rat MI tissue homogenate

Four hiPSC lines (hiPSC-A, B, C and D; detailed identification is found in Methods) were used in this study, each were confirmed to express surface and intracellular pluripotency markers and were karyotypically normal (Fig. S1). To generate hiPSC-CMs, we utilised the well-established protocol published by Burridge et al. [4] or a commercial CM Differentiation Kit (STEMCELL Technologies) as an alternative. Spontaneous cell contractions were observed between days 7 and 11 post-induction of differentiation (Vid. S1).

Cardiomyocyte identity and differentiation efficiency were validated using flow cytometry, RT-PCR, and immunofluorescence staining. The Burridge protocol consistently yielded cTnT-positive cells across all four hiPSC lines (Fig. S2A), with protein expression of cTnT, NKX2.5, and α-actinin confirmed at day 15 (Fig. S2B). Gene expression profiling showed downregulation of pluripotency markers (POU5F1, NANOG, SOX2), transient upregulation of early mesoderm markers (MESP1, KIT), and increased expression of cardiomyocyte markers (NKX2-5, TNNT2, MYL7, MYL2) over time (Fig. S2C–G). For all downstream experiments, only differentiations achieving > 60% cTnT + cells by flow cytometry were included.

**Fig. 1:**
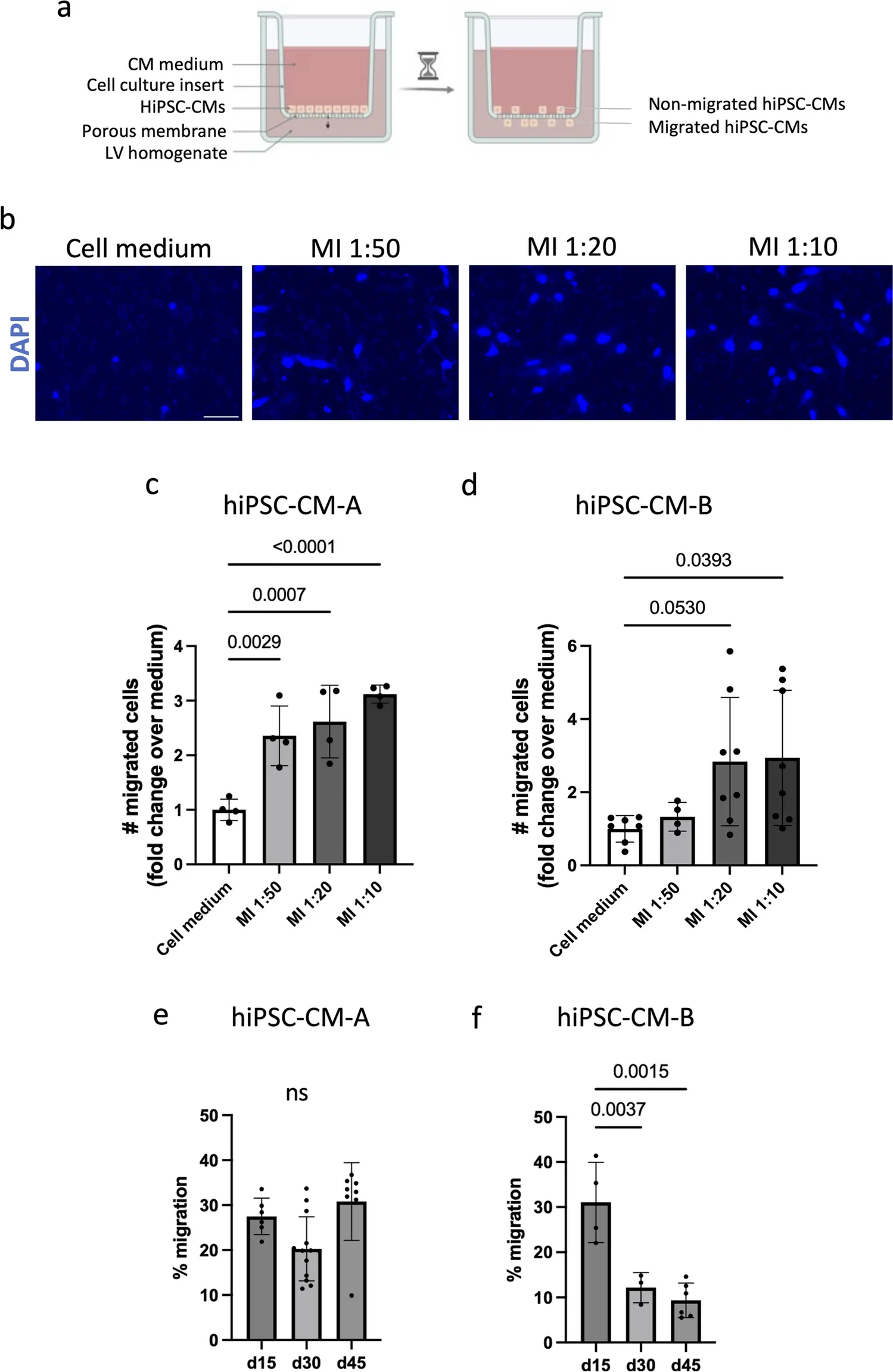
HiPSC-CMs exhibit a concentration-dependent chemotactic response to rat MI tissue homogenate. **a** Schematic of the Transwell migration assay. **b** Human IPSC-CMS were seeded onto transwells, exposed to cell medium alone, non-MI, or MI homogenate at varying dilutions for 24 h. Non-migrated hiPSC-CMS on the upper surface of the membrane were removed by swabbing and migrated hiPSC-CMs on the lower surface were stained with DAPI and imaged. Scale bar = 50 μm. **c**–**d** Quantification of migrated nuclei for hiPSC-CM-A and hiPSC-CM-B, respectively. Bars represent the mean number of nuclei counted across 4–8 wells per condition. N = 2. **e–f** Quantification of migrated nuclei for hiPSC-CM-A and hiPSC-CM-B seeded at different stages of differentiation and incubated with MI homogenate (1:10 dilution) for 24 h. Bars represent the mean number of **e**) hiPSC-CM-A nuclei counted across 6–10 wells per condition (*N* = 1–3) and **f**) Human IPSC-CM-B, 3–6 wells per condition (*N* = 1–2). Statistical analyses were performed using one-way ANOVA with Dunnett’s correction. Bars represent mean ± SD

To assess whether MI tissue exerts a chemotactic effect on hiPSC-CMs, we developed a Transwell migration (TWM) assay (Fig. 1A). Human iPSC-CM-A cells were seeded in the upper chamber and exposed to various dilutions of MI homogenate, derived from rat left-ventricular tissue harvested 1 day post-MI. After 24 h, migrated cells on the lower membrane surface were stained with DAPI and quantified (Fig. 1B–D). A significant, concentration-dependent increase in hiPSC-CM-A migration was observed in response to MI homogenate compared to control medium, with a peak threefold increase at a 1:10 dilution (Fig. 1C). Similarly, hiPSC-CM-B showed a robust threefold increase at the same dilution (Fig. 1D), and comparable results were seen in hiPSC-CM-C (Fig. S3).

We next explored whether the duration of hiPSC-CM culture influences their migratory capacity, given the known association between cardiomyocyte maturation and reduced motility [27]. Cells were assessed at various differentiation time points (day 15 to day 45) in the TWM assay. For hiPSC-CM-A, migration remained stable (~ 20–30%) across this time frame, with no statistically significant changes (Fig. 1E). In contrast, hiPSC-CM-B displayed a time-dependent decline in migration, decreasing from 28% at day 15 to just 9% by day 45 (Fig. 1F). These findings suggest cell line-specific differences in how prolonged culture impacts migratory ability.

Our TWM assays demonstrate that rat MI tissue homogenate induces a strong, concentration-dependent chemotactic response in hiPSC-CMs across multiple cell lines. Furthermore, cell line-specific and temporal factors influence the persistence of this migratory capacity during extended in vitro culture. These findings underscore the importance of accounting for both biological variability and maturation state when considering hiPSC-CMs for regenerative applications.

### An MI stimulus accelerates collective hiPSC-CM migration

**Fig. 2:**
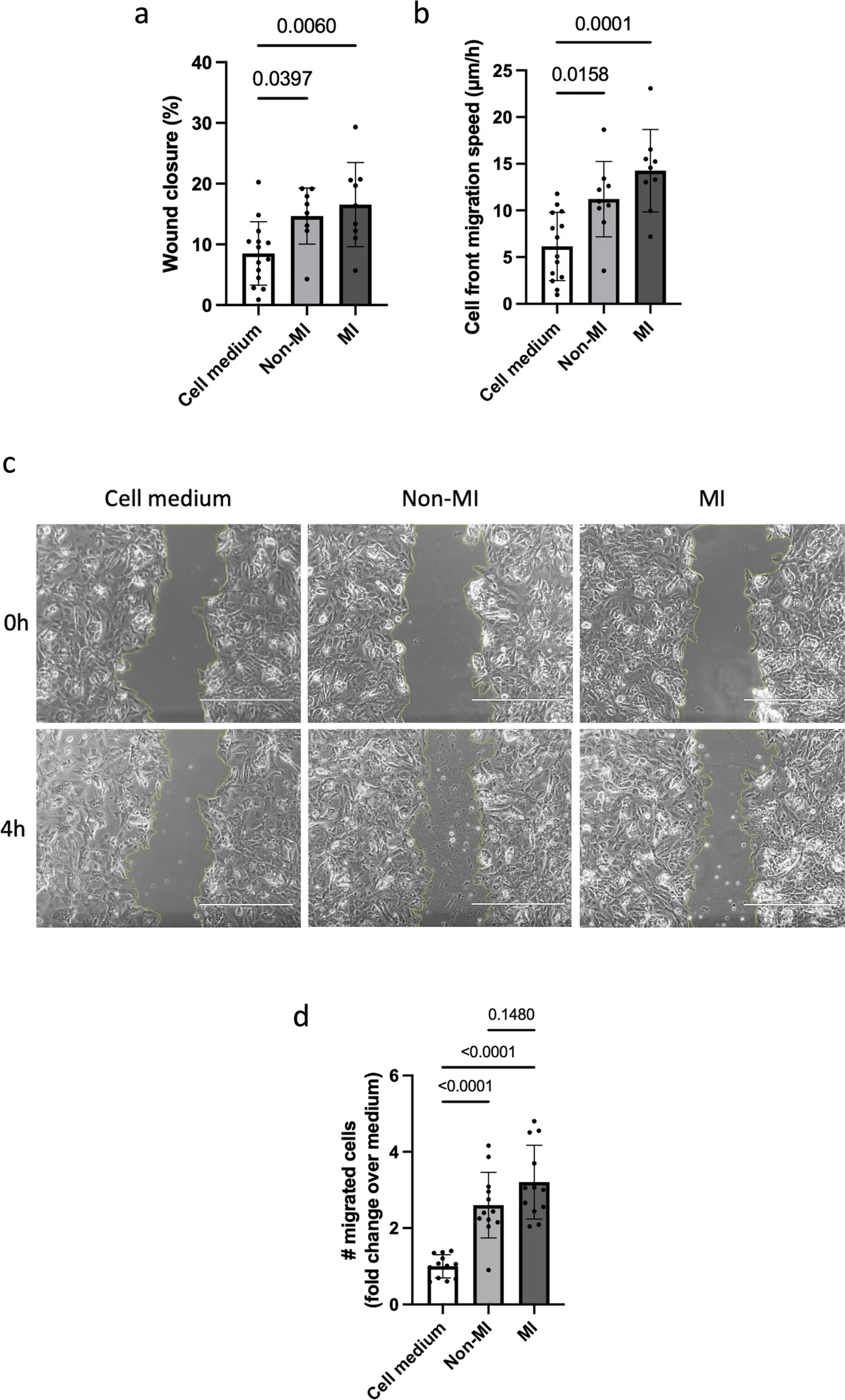
MI stimulus accelerates collective hiPSC-CM migration. Human IPSC-CM-D (commercial kit-derived) were seeded to form a confluent monolayer and a scratch was generated. Cells were incubated for 4 h in culture medium alone (*n* =14) or medium supplemented with MI homogenate (1:10 dilution; *n* = 9) or non-MI homogenate (1:10 dilution; *n* = 9). **a**) Wound closure and **b**) cell front migration speed were quantified after 4 h of incubation, *N*=2. **c** Representative images of day 15 hiPSC-CM-D in the scratch wound assay. Scale bar= 1000 μm. **d**) Human IPSC-CM-A were seeded on Transwell inserts and exposed to a 1:20 dilution of MI or non-MI homogenate for 4 h (*n*=12). Technical replicates are indicated with ‘*n*’ and biological replication with ‘*N*’. Statistical analyses were performed using a one-way ANOVA followed by Tukey’s pairwise comparisons. Bars represent mean ± SD

To further investigate the impact of MI-associated cues on hiPSC-CM migration, we employed a wound-healing assay to assess collective migration and wound closure dynamics. Human iPSC-CM-D monolayers were incubated with LV tissue homogenate from either MI or non-MI (sham) rats, and wound closure was assessed 4 h after scratch injury (Fig. 2A–C).

Exposure to MI homogenate (1:10 dilution) significantly enhanced wound closure compared to control medium (16.6 ± 6.9% vs. 8.5 ± 5.2%, respectively). This was accompanied by a substantial increase in the cell front migration speed, which more than doubled from 6.2 ± 3.7 μm/h in control conditions to 14.3 ± 4.4 μm/h in the presence of MI homogenate. Our observed migration speeds closely match those reported for hESC-CMs [30], reinforcing the biological validity of the model.

Interestingly, exposure to non-MI (sham) LV homogenate also resulted in enhanced wound closure (14.7 ± 4.6%) and increased migration speed (11.2 ± 4.0 μm/h) (Fig. 2A–C), though both metrics remained lower than those observed with MI homogenate. To further explore this observation, we conducted a TWM assay. Consistent with the wound-healing results, non-MI homogenate induced a significant increase in hiPSC-CM migration relative to medium alone, but to a lesser extent than MI homogenate (Fig. 2D). This suggests that whilst non-MI homogenate does have chemokinetic effects, likely due to intracellular content release during tissue homogenisation, it lacks the full complement of injury-associated chemotactic cues present in the MI tissue.

### Migrated hiPSC-CMs exhibit a differential gene expression profile from non-migrated hiPSC-CMs

To identify pathways involved in hiPSC-CM migration, we employed a TWM assay in which day 15 hiPSC-CMs were exposed to rat MI homogenate for 24 h. Migrated cells were collected from the lower surface of the membrane, whilst non-migrated cells were harvested from the upper surface. RNA was extracted from both populations for bulk RNA sequencing to comprehensively profile gene expression differences.

To confirm cardiomyocyte identity, expression of canonical CM markers (TNNT2, NKX2-5, MYL7) was examined, showing comparable levels between migrated and non-migrated groups in both cell lines (Table S1). CM subtype markers indicated a heterogeneous population consisting of atrial (NR2F2), ventricular (MYL2, IRX4), and nodal (TBX18, HCN4) subtypes, consistent with expectations from the Burridge differentiation protocol (Fig. S2, Table S1). The immature phenotype of hiPSC-CMs was reflected by higher expression of foetal isoforms TNNI1 and MYH6 relative to adult isoforms TNNI3 and MYH7. Evaluation of potential contaminating cell types revealed low expression of cardiac fibroblast markers DDR2 and CD90 (THY1), and absence of endothelial marker CD31 in both lines (Table S1).

**Fig. 3:**
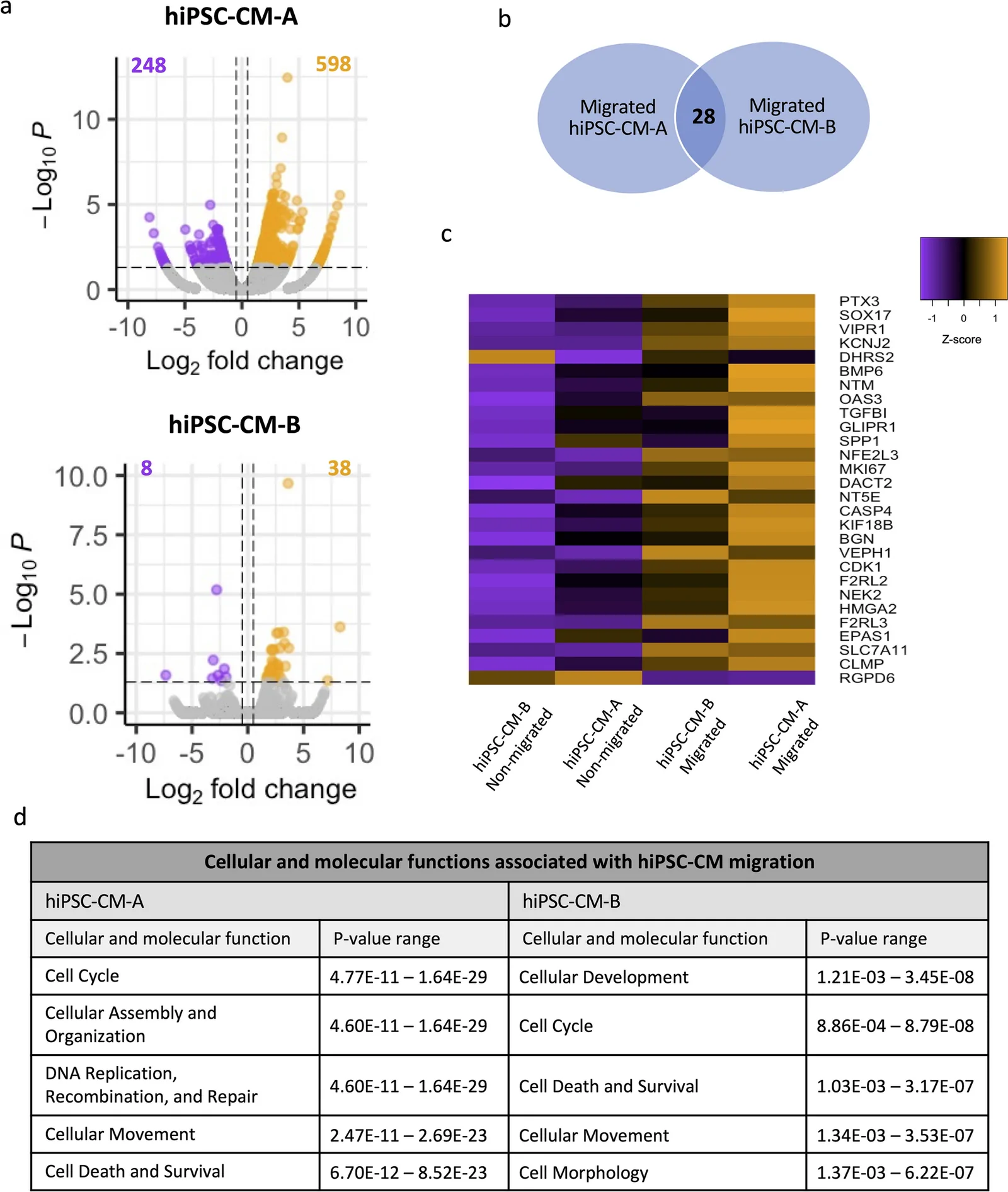
Migrated hiPSC-CMS display a distinct gene expression profile relative to non-migrated hiPSC-CMs. HIPSC-CM-A and hiPSC-CM-B were seeded on transwell inserts. Following overnight attachment, hiPSC-CMs were exposed to MI homogenate (1:10 dilution) for 24 h. Migrated and non-migrated cells were harvested separately and analysed by bulk RNAseq. **a** Differential gene expression analysis of migrated cells versus non-migrated cells from hiPSC-CM-A and hiPSC-CM-B. Significantly upregulated and downregulated genes are shown in orange and purple, respectively, whilst non-significant genes are shown in grey. Differentially expressed genes were defined by |log2 fold change| > 1 and adjusted *p* value (false discovery rate, FDR) <0.05. **b–c** Integration of the hiPSC-CM-A and hiPSC-CM-B datasets identified a panel of 28 genes shared by migrated populations. **d** Cellular and molecular functions associated with hiPSC-CM migration identified via ingenuity pathway analysis

Following 24 h incubation with MI homogenate, differential gene expression analysis identified 849 genes significantly altered in migrated versus non-migrated hiPSC-CM-A cells (598 upregulated and 248 downregulated; adjusted *p* value < 0.05, log2 fold change > 1) (Fig. 3A). In contrast, only 46 genes were significantly differentially expressed in hiPSC-CM-B (38 upregulated, 8 downregulated). Integrating data from both lines yielded a panel of 28 differentially expressed genes (DEGs) (Fig. 3B–C). Interestingly, proliferative markers MKI67 and CDK1 were present in this panel, suggesting that migrated hiPSC-CMs in both lines were more proliferative then non-migrating hiPSC-CMs.

From this panel, 14 genes of interest were selected for validation by RT-qPCR across hiPSC-CM-A, -B, and -C lines, confirming reproducible upregulation (Fig. S4). Gene Ontology (GO) enrichment analysis revealed that DEGs were predominantly associated with extracellular matrix organization, cell migration, and chemotaxis (Table S2). Ingenuity Pathway Analysis (IPA, QIAGEN) further identified cellular movement as a top molecular and cellular function linked to the differentially expressed genes in both datasets (Fig. 3D), supporting the ability of our model to uncover key regulators of hiPSC-CM migration.

### Inhibition of TGF-β signalling reduces MI homogenate-induced hiPSC-CM migration

To investigate potential upstream regulators driving hiPSC-CM migration, we examined links between DEGs identified in our RNA-seq datasets. Amongst the 28 DEGs shared between migrated hiPSC-CM-A and hiPSC-CM-B, seven genes (SOX17, BMP6, TGFBI, SPP1, NT5E, HMGA2, and DACT2) have previously been directly associated with TGF-β signalling pathways [6, 10, 16, 22, 23, 38]. Furthermore, Ingenuity Pathway Analysis (IPA) identified TGF-β1 as a central upstream regulator (z-score: 2.195, *p* value = 4.27 × 10^–6^) associated with 12 of the 28 DEGs, implicating it in the observed migratory behaviour.

TGF-β signalling is known to be upregulated in the myocardium following MI [24, 40] and plays a crucial role in heart regeneration in the zebrafish model, particularly by inducing an epithelial-to-mesenchymal transition (EMT)-like response in cardiomyocytes, which promotes migratory activity [33]. Notably, HMGA2, one of the DEGs identified in our study, is a well-characterised mediator of TGF-β-induced EMT [38].

**Fig. 4:**
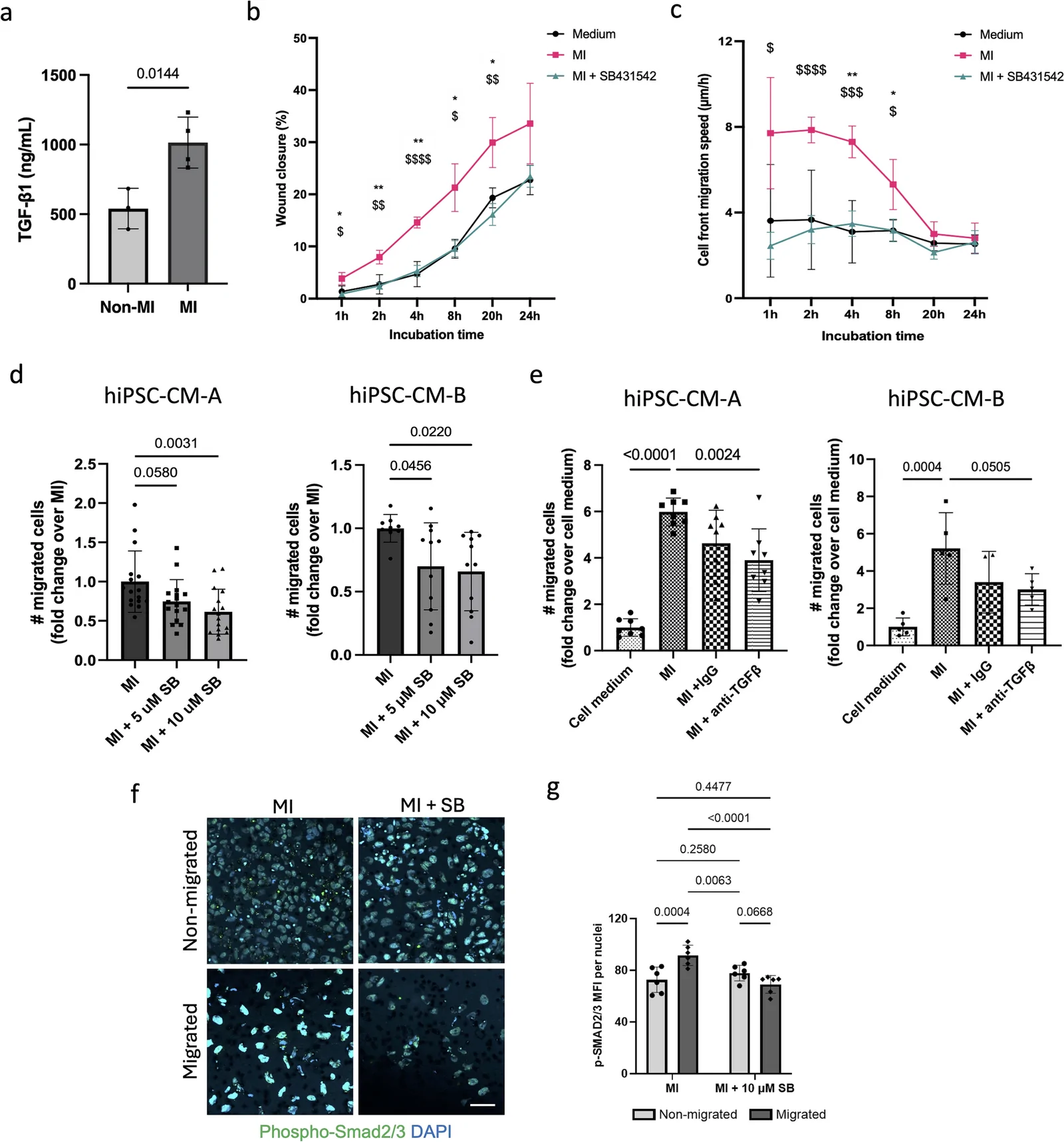
Inhibition of TGF-ẞ signalling reduces MI homogenate-induced hiPSC-CM migration. **a** TGF-β1 levels in non-MI (*n* = 3) and MI (*n* = 4) left-ventricular (LV) homogenate samples were quantified by ELISA. **b–c** Day 15 hiPSC-CM-A were seeded overnight, after which a scratch wound was generated. Cells were then incubated for 24 h with culture medium alone, MI homogenate (1:20 dilution), or MI homogenate (1:20) supplemented with 10 μM SB431542. **b** Wound closure and **c** cell front migration speed were quantified over 4 wells *: P<0.05, **: *P*<0.01, MI vs. medium, $: *P*<0.05, $$: *P*<0.01, $$$: *P*<0.001, $$$$: *P*<0.0001, MI vs. MI + SB431542. **d** hiPSC-CMs were seeded on transwell inserts and exposed to MI homogenate (1:20 dilution) with or without a 15 min pre-incubation of SB431542 (5 μM or 10 μM) for 4 h. Migrated cells were quantified in hiPSC-CM-A (16 wells, *N* = 2) and hiPSC-CM-B (10–11 wells, *N* = 3). **e** hiPSC-CMs were seeded on Transwell inserts and incubated for 4 h with culture medium, MI homogenate (1:20), or MI homogenate supplemented with 20 μg/mL IgG or TGF-β neutralising antibody. Quantification was performed in hiPSC-CM-A (12 wells) and hiPSC-CM-B (4–5 wells). **f–g** hiPSC-CM-A were seeded overnight, exposed to MI homogenate (1:10) or MI homogenate (1:10) supplemented with 10 μM SB431542 for 24 h. Both migrated and non-migrated hiPSC-CMs were stained for phospho-Smad2/3 and DAPI and imaged. Scale bar = 50 μm. Quantification was performed across 6 wells per condition (*N* = 1–2). Statistical analyses were performed using a two-tailed unpaired t test (**a**), two-way ANOVA followed by Tukey’s multiple comparisons test (**b**, **c**), one-way ANOVA followed by Dunnett's correction (**d**, **e**), or Tukey’s multiple comparisons test (**g**). Bars represent mean ± SD

We confirmed the presence of TGF-β1 protein in the MI homogenate, measuring 1014 ± 183 ng/mL, which was significantly higher than in non-MI homogenate (540 ± 146 ng/mL, Fig. 4A). The detectable level of TGF-β1 in non-MI homogenate may account for its observed partial chemokinetic and chemotactic effects on hiPSC-CM migration (as described in Fig. 2).

To confirm activation of TGF-β signalling, we exposed hiPSC-CM-A to MI homogenate (1:20) and performed immunostaining for phosphorylated Smad2/3 after 24 h of incubation (Fig. 4B, C). We observed positive staining in both migrated and non-migrated cells; however, signal intensity was significantly higher in the migrated population, suggesting that TGF-β signalling is associated with and may contribute to hiPSC-CM migration (Fig. 4C).

To further investigate this, we pre-treated the hiPSC-CMs with the pharmacological inhibitor SB431542, which selectively blocks the kinase activity of the TGF-β type I receptor (ALK5) [17]. Upon inhibition of TGF-β signalling, we observed a reduction in cellular migration as well as a significant reduction in phosphorylated Smad2/3 expression, supporting a functional role for TGF-β signalling in regulating hiPSC-CM migratory behaviour (Fig. 4B, C).

Alongside upregulation of genes associated with TGF-β signalling, bulk RNA-seq also showed increased expression of TGF-β response genes in migrated versus non-migrated hiPSC-CMs (Fig. 4D). SMAD3 was shown to be upregulated, which is typical following injury or disease, whilst SMAD2 levels typically remain stable or are actively downregulated. This is because the TGFB–SMAD3 complex directly binds to the *SMAD3* promoter region, creating a feed-forward amplification loop, whilst SMAD2 acts as a negative regulator of this auto-induction loop.

To further assess the functional relevance of TGF-β signalling in hiPSC-CM migration, we performed a scratch wound assay with SB431542 treatment from 1 to 20 h post-incubation with MI homogenate significantly reduced wound closure compared to MI homogenate alone (Fig. 4E). Similarly, cell front migration velocity was markedly decreased in the presence of SB431542, returning to baseline levels observed in control medium (Fig. 4F). Notably, the most pronounced effect occurred during the first 8 h of incubation, suggesting that TGF-β signalling plays an early and critical role in initiating hiPSC-CM migration.

To further confirm the involvement of TGF-β signalling, we performed a TWM assay with inhibition of TGF-β. After 4 h of incubation, the addition 10 μM SB431542 to MI homogenate significantly reduced the number of migrated hiPSC-CMs (Fig. 4G, H). Similarly, the application of a neutralising anti–TGF-β antibody attenuated MI homogenate-induced migration. In hiPSC-CM-A, neutralisation led to a significant reduction in migrated cells (Fig. 4I), whilst hiPSC-CM-B showed a strong decreasing trend (*p* = 0.05, Fig. 4J).

These findings provide robust evidence that TGF-β is a key mediator of the migratory response of hiPSC-CMs to MI tissue stimuli. Pharmacological inhibition and antibody-based neutralisation both significantly impaired migration, supporting a causal role for TGF-β signalling in promoting hiPSC-CM motility. These results highlight TGF-β as a potential therapeutic target to modulate transplanted cell behaviour in cardiac regeneration strategies.

## Discussion

This study provides, to our knowledge, the first comprehensive characterisation of the migratory capacity of hiPSC-CMs in response to factors derived from infarcted myocardium. Using two complementary gold-standard in vitro migration models, Transwell migration (TWM), and scratch wound assays, we demonstrated that hiPSC-CMs exhibit concentration-dependent migratory responses following exposure to rat MI tissue homogenate. Importantly, by employing multiple independent hiPSC-CM lines, we identified substantial donor-dependent differences in migratory behaviour, with one line consistently displaying greater responsiveness to MI-derived signals. These findings establish CM migration towards infarct-associated cues as a previously underappreciated but biologically relevant property of hiPSC-CMs and highlight intrinsic variability between human cell lines as a potentially important determinant of regenerative efficacy.

The TWM model enabled physical separation of migrated and non-migrated hiPSC-CM populations, facilitating bulk RNA sequencing to identify molecular pathways associated with migration. Amongst the 28 DEGs shared across both hiPSC-CM lines, several were linked to TGF-β signalling, which emerged through an unbiased transcriptomic and pathway analysis approach as a dominant candidate regulator of migration. Although TGF-β has previously been implicated as a chemoattractant [35, 41] and is known to regulate migration across multiple cell types [26, 28, 44], including during cardiac development and repair [1], our findings extend this knowledge by demonstrating that human hiPSC-CMs respond to infarct-derived signals through a conserved TGF-β-dependent migratory mechanism. In regenerative species such as zebrafish, TGF-β signalling has been shown to mediate CM migration during cardiac regeneration through an EMT-like process [7]. Our data support the concept that this migratory signalling axis is evolutionarily conserved and remains functionally active in human hiPSC-CMs exposed to post-MI cues.

We demonstrated that MI homogenate contained significantly higher levels of TGF-β1 than sham homogenate, supporting its relevance within the post-infarct microenvironment. However, tissue homogenisation may promote activation of latent TGF-β complexes, potentially inflating measured levels of active TGF-β relative to the in vivo setting. We acknowledge that this remains speculative in the absence of direct in vivo validation. Additionally, whilst IPA specifically implicated TGF-β1, the use of a pan-TGF-β neutralising antibody does not exclude potential contributions from other TGF-β family members.

The observation that only a subset of cells migrated despite widespread Smad2/3 activation supports the concept that hiPSC-CM migration is regulated by a combination of signalling pathways and cellular intrinsic factors rather than a single chemotactic stimulus. However, in the absence of TGF-β (cell culture media only or TGF-β inhibition), Smad2/3 remains inactivated and migration is limited. These results suggest a critical role for TGF-β signalling as an important component of the post-infarct microenvironment that may facilitate, but does not solely determine, hiPSC-CM migratory behaviour.

Functional validation experiments demonstrated that inhibition of TGF-β signalling using either SB431542 or a TGF-β neutralising antibody significantly impaired hiPSC-CM migration. These findings confirm that TGF-β signalling is not merely associated with migration but actively contributes to the migratory response of hiPSC-CMs towards infarct-derived stimuli. Importantly, our findings do not suggest that exogenous TGF-β administration would be therapeutically desirable, particularly given the well-established profibrotic effects of TGF-β signalling in the injured heart [11, 12, 32, 43]. Rather, our data raise the possibility that enhancing the intrinsic responsiveness of transplanted CMs to endogenous injury-associated TGF-β gradients may improve their migratory and engraftment potential. Nevertheless, the highly context-dependent nature of TGF-β signalling, including its beneficial and deleterious effects depending on injury phase and target cell type, will require careful consideration in future translational studies.

Interestingly, transcriptomic analysis also identified a proliferative gene expression signature in migrated hiPSC-CMs, including upregulation of MKI67 and CDK1. This observation suggests that migrated cells may possess enhanced proliferative potential, raising the possibility that migratory and proliferative phenotypes are linked. If confirmed in vivo, such properties could provide dual therapeutic benefits by promoting both cell engraftment and remuscularisation of infarcted myocardium. Further investigation will be required to determine whether these transcriptional changes translate into meaningful proliferative activity after transplantation.

We also examined the relationship between hiPSC-CM maturation and migratory capacity. As expected, prolonged culture was associated with either stable or reduced migration, depending on the cell line examined. Whilst hiPSC-CM-A maintained migratory capacity up to day 45 of differentiation, hiPSC-CM-B demonstrated a marked decline over time, further emphasising the importance of cell line-intrinsic properties in determining migratory behaviour. These findings suggest that both maturation state and donor-specific biology influence migration potential. Defining the optimal differentiation stage for transplantation, therefore, remains an important challenge, particularly as hiPSC-CMs undergo substantial metabolic, electrophysiological, and stress-response remodelling during maturation, all of which may affect therapeutic performance.

Our findings also raise important questions regarding subtype-specific migration. Although expression of the atrial-associated marker MLC2a declined over time, whilst ventricular-associated MLC2v increased, consistent with progressive maturation, we observed no significant differences in MYL2 or MYL7 expression between migrated and non-migrated populations. These results suggest that both atrial- and ventricular-like hiPSC-CMs, as well as intermediate populations, retain the capacity to respond to migratory cues. However, recently developed differentiation protocols capable of generating highly enriched ventricular hiPSC-CM populations [9] may enable more refined investigation of subtype-dependent migratory responses in future studies.

In summary, this study identifies migration towards damaged myocardium-derived signals as a previously under recognised property of human hiPSC-CMs and establishes TGF-β signalling as a key regulator of this response in a human cell-based model. Beyond confirming prior observations from regenerative animal models, our work demonstrates that this pathway is conserved in human CMs and reveals substantial donor-dependent variability in migratory behaviour. Together, these findings provide new insight into how transplanted hiPSC-CMs may interact with the post-MI microenvironment and establish a framework for future studies aimed at improving cardiomyocyte engraftment and integration during cardiac regeneration.

## Materials and methods

### Human iPSC culture

Four hiPSC lines [KUXP (WTSIi008-A), QOLG (WTSIi004-B), ROBP (WTSIi018-A), and KOLF (WTSIi090-A)] were obtained from the European Bank for Induced Pluripotent Stem Cells (EBiSC) and maintained in feeder-free, serum-free 2D culture at 37 °C and 5% CO_2_. For CM differentiation, hiPSCs were grown in E8 Flex medium (Thermofisher, A2858501) on recombinant human vitronectin (Thermofisher, A14700)-coated plates for 3–4 days until reaching approximately ~ 85% confluency. Differentiation into cardiomyocytes was initiated using a protocol adapted from Burridge et al. [4] Briefly, hiPSCs were incubated in CDM3 medium; RPMI 1640 supplemented with 75 mg/mL recombinant human albumin (Merck, A7931) and 64 mg/mL L-ascorbic acid 2-phosphate (Merck, 49752) with the addition of 6 μM CHIR99021 (Cambridge Bioscience, CAY13122) for 48 h. On day 2, the medium was replaced with CDM3 containing 2 μM Wnt-C59 (Selleckchem, S7037), and from day 4 onwards, cells were maintained in CDM3 alone, with media refreshed every two days. Alternatively, where indicated, hiPSC-CMs were generated using a commercially available Cardiomyocyte Differentiation Kit (Thermofisher, A2921201), following the manufacturer’s instructions.

### Heart tissue homogenisation

Adult male Sprague–Dawley rats (8–10 weeks) were purchased from Charles River UK and underwent either MI induction via left coronary artery (LCA) ligation or a sham surgical procedure, as described previously [20]. For MI, the LCA was ligated at the level of the lower edge of the left atrial appendage via left thoracotomy under isoflurane anaesthesia and mechanical ventilation. Successful induction of ischaemia was confirmed by regional epicardial colour change and the presence of dyskinesia. Following chest and skin closure, animals were extubated and allowed to recover in their cage. Sham-operated animals underwent an open-chest procedure without LCA ligation. All animals were sacrificed 24 h post-surgery. The left-ventricular (LV) wall was then harvested, either freshly homogenised or snap-frozen in liquid nitrogen and stored at –80 °C for later use. For each individual experiment, one LV was used per homogenate.

Tissue homogenisation was performed PBS supplemented with protease inhibitors (cOmplete^™^, EDTA-free Protease Inhibitor Cocktail; Sigma-Aldrich, 11,873,580,001) using a Precellys 24 tissue homogeniser. Homogenates were centrifuged at 6000 × g for 15 min, and the supernatant was collected for immediate use or stored at –80 °C for subsequent experiments.

All animal procedures were approved by the institutional ethics committee at Queen Mary University of London and the UK Home Office. Experiments conformed to the *Principles of Laboratory Animal Care* (National Society for Medical Research) and the *Guide for the Care and Use of Laboratory Animals* (U.S. National Institutes of Health, 1996).

### Wound-healing assays

Human iPSC-CMs were seeded at a density of ≥ 2.5 × 10^5^ cells/cm^2^ to form a confluent monolayer. To promote cell attachment, hiPSC-CMs were incubated overnight with 10 μM Y-27632 (Rock inhibitor; Cambridge BioScience, HY-10071-5 mg). The following day, a scratch wound was generated across the hiPSC-CM monolayer using a sterile P1000 tip. After washing with PBS to remove detached cells, hiPSC-CMs were incubated for up to 24 h with either cell media alone or medium supplemented with LV homogenate from MI or sham-operated rat hearts (diluted as specified).

Cell migration was assessed by capturing images at defined time points using either a Keyence BZ-X810 microscope or an EVOS XL Core Cell Imaging System. Quantitative analysis was performed using ImageJ software. Percentage wound closure was calculated as follows:

$$\begin{aligned} &\frac{wound\, area\, at\, 4h\, \left({\upmu \mathrm{m}}^{2}\right)-initial\, wound\, area \,\left({\upmu \mathrm{m}}^{2}\right)}{initial \,wound\, area\, \left({\upmu \mathrm{m}}^{2}\right) } \\ &\quad \times 100\end{aligned}$$

Cell front migration was calculated as follows;

$$\begin{aligned}\frac{wound\, area\, at\, 4h \,\left({\upmu \mathrm{m}}^{2}\right)-initial\, wound\, area\, \left({\upmu \mathrm{m}}^{2}\right)}{height\, of\, the\, image\, \left(\upmu \mathrm{m}\right)},\end{aligned}$$

in which the height of the image represents the y-axis, to obtain the difference in horizontal distance (x-axis), i.e., the cell front migration (μm). This number is then divided by the incubation time to obtain the cell front migration speed (μm/h). For the TGF-β inhibition experiment (Fig. 4), 10 μM SB431542 (Tocris, 1614/1) was supplemented to the media.

### Transwell migration assays

All TWM assays were performed in 24-well plates. Transwell inserts with 8 μM pore size (Starstedt, 83.3932.800) were pre-coated on both sides with ~ 22 μg/cm^2^ Geltrex Ready to Use (Thermofisher, A1569601) for 1 h at 37 °C. After aspiration of the residual coating, 1 × 10^5^ hiPSC-CMs per insert were seeded into the upper chamber in cardiomyocyte support medium supplemented with 10 μM Y-27632 and incubated overnight to allow cell attachment. The next day, the medium in the lower chamber was replaced with cell medium alone or medium supplemented with LV homogenate from MI or sham-operated rat hearts at the indicated dilution. After 24 h of incubation, cells were fixed in 4% paraformaldehyde for 15 min at room temperature (RT) followed by a PBS wash and incubation with DAPI for 15 min at RT. To ensure that only migrated cells on the lower surface were visualised, the upper surface of the insert was gently swabbed with a cotton applicator to remove non-migrated cells. The membranes were then carefully excised and mounted onto glass microscope slides. Images were acquired on a Keyence BZ-X180 or Zeiss LSM800 microscope. Quantification of migrated cells was performed using the ImageJ software. For the TGF-β inhibition experiments, 10 μM SB431542 (Tocris, 1614/1) was supplemented to the upper insert 10 min prior to their incubation with medium and LV homogenate. For the TGF-β neutralisation studies, we used an anti-TGF-β−1,2,3 antibody (1D11.16.8, Thermofisher, 16–9243-85) or an IgG isotype control at a concentration of 20 μg/mL.

### ELISA

TGF-β1 levels were measured in homogenates from sham and MI tissues using a TGF-beta 1 Quantikine ELISA kit (R and D, Cat #DB100C). To activate latent TGF-β1, tissue supernatants were incubated with 1 M HCl for 10 min, followed by neutralisation with 1.2 M NaOH/0.5 M HEPES. Activated samples or standards were added to wells pre-coated with TGF-β1 capture antibody, along with the assay diluent. After a 2 h incubation at RT, plates were washed four times before addition of the TGF-β1 conjugate. Plates were then incubated for a further 2 h, followed by four washes. Substrate solution was then added and incubated for 30 min at RT in the dark. The reaction was stopped by the addition of Stop Solution and absorbance was measured at 450 nm with wavelength correction at 540 nm using a microplate reader.

### Bulk RNA-seq

Human iPSCs were seeded onto Transwell inserts at day 15 of CM differentiation, as previously described. After 24 h of incubation with a 1:10 dilution of left-ventricular homogenate from an MI rat heart in the lower compartment, culture media was aspirated and the inserts washed in PBS. To isolate migrating and non-migrating cells, the upper and lower surfaces of the inserts were swabbed separately.

RNA was extracted by lysing the cells directly on the inserts using RLT lysis buffer from the Qiagen RNeasy mini kit (Qiagen, 74,104), following the manufacturer’s instructions. Samples with RNA integrity numbers (RIN) ≥ 9.4 and a minimum input of 100 ng of total RNA were used for bulk RNA-seq (20 million reads per samples). Library preparation and paired-end 150 bp sequencing (PE150) were performed by Novogene Ltd, who also conducted downstream data analysis.

### Immunocytochemistry

Human iPSCs were harvested by incubation with EDTA for 3–5 min, followed by gentle detachment with culture media. Cells were seeded onto 16 mm coverslips in 12 well plates pre-coated with recombinant human vitronectin and cultured for 2–4 days before fixation with 4% paraformaldehyde in PBS for 15 min at 37 °C.

Ahead of immunostaining, hiPSC-CMs were replated overnight using the CM dissociation kit (StemCell Technologies, 05025). Briefly, wells were washed twice with PBS before incubation with CM dissociation medium (StemCell Technologies, 05026) at 37 °C for 10–15 min. Detached cells were collected using a 10 mL serological pipette and transferred into tubes containing CM support medium (StemCell Technologies, 05027). After centrifugation at 300 g for 5 min, the supernatant was removed, and the cell pellet was resuspended in CM support medium supplemented with 5 µM Y-27632. Cells were seeded into a new vitronectin-coated plate at the desired density (≥ 2.5 × 10^5^ cells/cm^2^) and allowed to attach overnight. The following day, cells were fixed with 4% paraformaldehyde in PBS for 15 min at 37 °C.

After fixation, hiPSCs and hiPSC-CMs were washed and permeabilised with 0.1% Triton-X-100 in PBS for 10 min at RT, then blocked using 10% donkey serum/PBS for 1 h at RT. Primary antibodies were applied overnight at 4 °C in the dark. After washing, cells were incubated with appropriate secondary antibodies for 30 min at 37 °C. DAPI was used as a nuclear counterstain. All wash steps were performed with 0.1% Tween in PBS. Imaging was conducted using a Zeiss LSM 800 or Keyence BZ-X810 microscope. The antibodies used for immunostaining can be found in Supplementary Tables S4 and S5.

For Fig. 4B, Transwell inserts were fixed, washed, permeabilised, blocked, and stained according to the above steps within the 24-well plates, and staining was performed with Phospho-SMAD2/3 (Thermofisher, PA5-110,155) at a 1:200 dilution.

### Flow cytometry

Human iPSCs were harvested by incubation with EDTA for 3–5 min followed by aspiration, followed by gentle detachment with culture media. Cells were then transferred to 15 mL tubes and centrifuged at 200 g for 5 min. The supernatant was aspirated, and cells were resuspended into single cells in filtered FACS buffer (PBS, 4% w/v bovine serum albumin (BSA), 10% v/v penicillin–streptomycin (Pen-Strep), 4% v/v EDTA). After repeating the last step, the cells were stained for 30 min at RT. The antibodies used can be found in Supplementary Tables S4–5.

HiPSC-CMs were dissociated with the Cardiomyocyte Dissociation Kit (see above) and stained with the Inside Stain Kit (Miltenyi Biotech, 130-090-477) according to the manufacturer’s protocol. Briefly, dissociated cells are counted with Trypan Blue and up to 1 million cells are washed by adding FACS buffer. After centrifugation at 200 g for 10 min, the cells are resuspended in 1:1 FACS buffer/Inside Fix buffer. After 20 min of incubation in the dark at RT, the cells are washed with FACS buffer and centrifuged at 200 g for 5 min. The washing step is repeated in Inside Perm. The cells were directly stained in Inside Perm buffer for 10–30 min at RT, or indirectly for 1 h at RT and with secondary antibodies for 30 min at RT in the dark. The antibodies used can be found in Supplementary Tables S4–5.

HiPSCs/hiPSC-CMs were subsequently washed and resuspended in incubation medium and were kept on ice until analysis. Data were acquired on a BD LSRFortessa instrument and analysed using FlowJo software.

### Real-time quantitative PCR (RT-qPCR)

Total RNA isolation was performed using the RNeasy mini kit (Qiagen) according to the manufacturer’s protocol. Briefly, harvested cells or heart homogenates were lysed in RLT lysis buffer for 5 min at RT. The resulting cell lysate was transferred to a QIAShredder column and was centrifuged at full speed for 3 min. Next, 70% ethanol was added to the lysate, which was transferred to an RNeasy silica column, followed by centrifugation at 1.2 × 10^4^ rpm for 15 s. This was followed by a washing step in RW1 wash buffer and a DNase I incubation for 15 min at RT, followed by another wash step with RW1 and twice with RPE, followed by dry centrifugation of the column at 1.2 × 10^4^ rpm for 1 min. The RNA was eluted in RNase free ddH2O for 2 min at RT followed by centrifugation at 1.2 × 10^4^ rpm for 1 min. Eluted RNA was kept on ice until further use or stored at −80 °C for future use. RNA was subsequently reverse transcribed into cDNA with the High-Capacity Reverse Transcription Kit (Thermofisher, 4,368,814) with the following thermal cycler settings: 10 min at 25 °C, 120 min at 37 °C, and 5 min at 85 °C. Gene expression was quantified by real-time PCR of 10 ng cDNA of each sample with the PowerUp SYBR Green supermix (Thermofisher, A257420). β-actin and GAPDH were used as housekeeping genes. Primers for each gene are given in Table S6.

The plate was transferred to a QuantStudio 7.0 Real-Time PCR machine (Applied Biosystems) and the following parameters were applied: 5 min 95 °C, 40 cycles of 15 s at 95 °C, 30 s at 60 °C, and 30 s at 72 °C, followed by a melt curve analysis from 55 to 95 °C.

### Statistics

All data were analysed using Graphpad Prism 9.0 software (Graphpad, San Diego, CA). Data are represented as mean ± standard deviation (SD). Statistical analysis was performed on data with a replicate number of 3 and above. Technical replicates are indicated with ‘*n*’ and biological replication with ‘*N*’, as detailed in the figure legends. For the comparison between two groups, two-tailed unpaired Student’s t tests were performed. Multiple group comparisons were performed with one-way or two-way analysis of variance (ANOVA) with Tukey’s post hoc test correction (3 groups) or Dunnett’s correction (> 3 groups). If the number of replicates was not equal between groups, a mixed-effects analysis was performed instead of an ANOVA, as the latter cannot deal with missing values. Statistical significance was accepted at *p* < 0.05.

## Supplementary Information

Below is the link to the electronic supplementary material.

Supplementary file1 (PDF 3905 KB)

Supplementary file2 (AVI 4542 KB)

## Data Availability

The RNA-seq data supporting the findings of this study have been deposited in the Gene Expression Omnibus (GEO) database and are publicly accessible under accession number GSE317615.

## References

[1] Azhar M, Schultz JEJ, Grupp I, Dorn GW, Meneton P, Molin DGM, Gittenberger-de Groot AC, Doetschman T (2003) Transforming growth factor beta in cardiovascular development and function. Cytokine Growth Factor Rev 14:391–407. 10.1016/S1359-6101(03)00044-3 12948523 PMC3855389

[2] Bergeron A, Brezai A, Shukr R, Villeneuve L, Allen BG, Qureshi WMS, Hentges KE, Calderone A (2021) Filamentous nestin and nonmuscle myosin IIB are associated with a migratory phenotype in neonatal rat cardiomyocytes. J Cell Physiol 236:1281–1294. 10.1002/jcp.29934 32654195

[3] Bock-Marquette I, Saxena A, White MD, DiMaio JM, Srivastava D (2004) Thymosin β4 activates integrin-linked kinase and promotes cardiac cell migration, survival and cardiac repair. Nature 432:466–472. 10.1038/nature03000 15565145

[4] Burridge PW, Holmström A, Wu JC (2015) Chemically defined culture and cardiomyocyte differentiation of human pluripotent stem cells. Curr Protoc Hum Genet 87:21.3.1-21.3.15. 10.1002/0471142905.hg2103s87 26439715 PMC4597313

[6] Cappelli C, Tellez A, Jara C, Alarcón S, Torres A, Mendoza P, Podestá L, Flores C, Quezada C, Oyarzún C, San Martín R (2020) The TGF-β profibrotic cascade targets ecto-5′-nucleotidase gene in proximal tubule epithelial cells and is a traceable marker of progressive diabetic kidney disease. Biochimica et Biophysica Acta (BBA) - Mol Basis Dis 1866:165796. 10.1016/j.bbadis.2020.165796 32289379

[7] Chablais F, Jaźwińska A (2012) The regenerative capacity of the zebrafish heart is dependent on TGFβ signaling. Development 139:1921–1930. 10.1242/dev.078543 22513374

[8] Cipriano G, Thum T, Weber N (2025) Exploring hiPSC-CM replacement therapy in ischemic hearts. Basic Res Cardiol. 10.1007/s00395-025-01117-w 40493218 PMC12325513

[9] Dark N, Cosson MV, Tsansizi LI, Owen TJ, Ferraro E, Francis AJ, Tsai S, Bouissou C, Weston A, Collinson L, Abi-Gerges N, Miller PE, MacLeod KT, Ehler E, Mitter R, Harding SE, Smith JC, Bernardo AS (2023) Generation of left ventricle-like cardiomyocytes with improved structural, functional, and metabolic maturity from human pluripotent stem cells. Cell Rep Methods 3:100456. 10.1016/j.crmeth.2023.100456 37159667 PMC10163040

[10] Dituri F, Cossu C, Mancarella S, Giannelli G (2019) The interactivity between TGFβ and BMP signaling in organogenesis, fibrosis, and cancer. Cells 8:1130. 10.3390/cells8101130 31547567 PMC6829314

[11] Dobaczewski M, Gonzalez-Quesada C, Frangogiannis NG (2010) The extracellular matrix as a modulator of the inflammatory and reparative response following myocardial infarction. J Mol Cell Cardiol 48:504–511. 10.1016/j.yjmcc.2009.07.015 19631653 PMC2824059

[12] Hanna A, Frangogiannis NG (2019) The role of the TGF-β superfamily in myocardial infarction. Front Cardiovasc Med 6:140. 10.3389/fcvm.2019.00140 31620450 PMC6760019

[13] Hata H, Bär A, Dorfman S, Vukadinovic Z, Sawa Y, Haverich A, Hilfiker A (2010) Engineering a novel three-dimensional contractile myocardial patch with cell sheets and decellularised matrix. Eur J Cardiothorac Surg 38:450–455. 10.1016/j.ejcts.2010.02.009 20335044

[14] Heusch G, Libby P, Gersh B, Yellon D, Böhm M, Lopaschuk G, Opie L (2014) Cardiovascular remodelling in coronary artery disease and heart failure. Lancet 383:1933–1943. 10.1016/S0140-6736(14)60107-0 24831770 PMC4330973

[15] Hosen MR, Militello G, Weirick T, Ponomareva Y, Dassanayaka S, Moore JB, Döring C, Wysoczynski M, Jones SP, Dimmeler S, Uchida S (2018) Airn regulates Igf2bp2 translation in cardiomyocytes. Circ Res 122:1347–1353. 10.1161/CIRCRESAHA.117.312215 29483092 PMC5948151

[16] Hou J, Huang S, Long Y, Huang J, Yang S, Yao J, Chen G, Yue Y, Liang M, Mei B, Li J, Wu Z (2020) DACT2 regulates structural and electrical atrial remodeling in atrial fibrillation. J Thorac Dis 12:2039–2048. 10.21037/jtd-19-4206 32642106 PMC7330378

[17] Inman GJ, Nicolás FJ, Callahan JF, Harling JD, Gaster LM, Reith AD, Laping NJ, Hill CS (2002) SB-431542 is a potent and specific inhibitor of TGF-β superfamily type I receptors ALK4, ALK5, and ALK7. Mol Pharmacol 62:65–74. 10.1124/mol.62.1.65 12065756

[18] Itou J, Oishi I, Kawakami H, Glass TJ, Richter J, Johnson A, Lund TC, Kawakami Y (2012) Migration of cardiomyocytes is essential for heart regeneration in zebrafish. Development 139:4133–4142. 10.1242/dev.079756 23034636

[19] Kawamura T, Ito Y, Ito E, Takeda M, Mikami T, Taguchi T, Mochizuki-Oda N, Sasai M, Shimamoto T, Nitta Y, Yoshioka D, Kawamura M, Kawamura A, Misumi Y, Sakata Y, Sawa Y, Miyagawa S (2023) Safety confirmation of iPSC-derived cardiomyocyte patch transplantation for ischemic cardiomyopathy. Front Cardiovasc Med 10:1182209. 10.3389/fcvm.2023.1182209 37781295 PMC10540447

[20] Kobayashi K, Ichihara Y, Sato N, Umeda N, Fields L, Fukumitsu M, Tago Y, Ito T, Kainuma S, Podaru M, Lewis-McDougall F, Yamahara K, Uppal R, Suzuki K (2019) On-site fabrication of bi-layered adhesive mesenchymal stromal cell dressings for heart failure. Biomaterials 209:41–53. 10.1016/j.biomaterials.2019.04.014 31026610 PMC6527869

[21] Kormos RL, Cowger J, Pagani FD, Teuteberg JJ, Goldstein DJ, Jacobs JP, Higgins RS, Stevenson LW, Stehlik J, Atluri P, Grady KL, Kirklin JK (2019) The Society of Thoracic Surgeons INTERMACS database annual report. Ann Thorac Surg 107:341–353. 10.1016/j.athoracsur.2018.11.011 30691584

[22] Kramerova I, Kumagai-Cress C, Ermolova N, Mokhonova N, Marinov M, Capote J, Becerra D, Quattrocelli M, Crosbie RH, Welch E, McNally EM, Spencer MJ (2019) Spp1 promotes TGFβ processing in fibroblasts. Hum Mol Genet 28:3431–3444. 10.1093/hmg/ddz181 31411676 PMC7345878

[23] Lange AW, Keiser AR, Wells JM, Zorn AM, Whitsett JA (2009) Sox17 promotes progenitor behavior and inhibits TGF-β/Smad3 signaling. PLoS ONE 4:e5711. 10.1371/journal.pone.0005711 19479035 PMC2682659

[24] Li Q, Xu Y, Li X, Guo Y, Liu G (2012) Inhibition of Rho-kinase ameliorates myocardial remodeling via TGF-β1-TAK1. Toxicol Lett 211:91–97. 10.1016/j.toxlet.2012.03.006 22465603

[26] Liu Z, Yi L, Du M, Gong G, Zhu Y (2019) TGF-β enhances migration via ERK/MAPK. Exp Ther Med 18:4457–4464. 10.3892/etm.2019.7522 PMC6509355 31105783

[27] Lundy SD, Zhu WZ, Regnier M, Laflamme MA (2013) Structural and functional maturation of PSC-derived cardiomyocytes. Stem Cells Dev 22:1991–2002. 10.1089/scd.2012.0490 23461462 PMC3699903

[28] Melzer C, von der Ohe J, Hass R, Ungefroren H (2017) TGF-β-dependent growth arrest and migration controlled by Rac1. Int J Mol Sci 18:1574. 10.3390/ijms18071574 28726720 PMC5536062

[29] Miki K, Uenaka H, Saito A, Miyagawa S, Sakaguchi T, Higuchi T, Shimizu T, Okano T, Yamanaka S, Sawa Y (2012) iPSC-derived myocardium improves cardiac function. Stem Cells Transl Med 1:430–437. 10.5966/sctm.2011-0038 23197822 PMC3659710

[30] Moyes KW, Sip CG, Obenza W, Yang E, Horst C, Welikson RE, Hauschka SD, Folch A, Laflamme MA (2013) Cardiomyocyte migration toward fibronectin and Wnt5a gradients. Stem Cells Dev 22:2315–2325. 10.1089/scd.2012.0586 23517131 PMC3732016

[31] National Institutes of Health (2023) Safety and efficacy of iPSC-derived engineered myocardium (NCT04396899). https://clinicaltrials.gov/study/NCT04396899

[32] Pardali E, ten Dijke P (2009) TGF-β signaling and tumor angiogenesis. Front Biosci 14:4848–4861. 10.2741/3573 19482591

[33] Peng Y, Wang W, Fang Y, Hu H, Chang N, Pang M, Hu YF, Li X, Long H, Xiong JW, Zhang R (2021) Inhibition of TGF-β/Smad3 disrupts cardiomyocyte proliferation during regeneration. Front Cell Dev Biol 9:632372. 10.3389/fcell.2021.632372 33816481 PMC8010688

[34] Qin X, Gao S, Yang Y, Wu L, Wang L (2019) MiR-25 promotes cardiomyocyte proliferation via Bim. J Cell Physiol 234:22103–22115. 10.1002/jcp.28773 31058341

[35] Reibman J, Meixler S, Lee TC, Gold LI, Cronstein BN, Haines KA, Kolasinski SL, Weissmann G (1991) TGF-β1 chemoattracts neutrophils. Proc Natl Acad Sci USA 88:6805–6809. 10.1073/pnas.88.15.6805 1650483 PMC52177

[36] Shiba Y, Gomibuchi T, Seto T, Wada Y, Ichimura H, Tanaka Y, Ogasawara T, Okada K, Shiba N, Sakamoto K, Ido D, Shiina T, Ohkura M, Nakai J, Uno N, Kazuki Y, Oshimura M, Minami I, Ikeda U (2016) IPSC-derived cardiomyocytes regenerate primate hearts. Nature 538:388–391. 10.1038/nature19815 27723741

[37] Slaughter MS, Rogers JG, Milano CA, Russell SD, Conte JV, Feldman D, Sun B, Tatooles AJ, Delgado RM, Long JW, Wozniak TC, Ghumman W, Farrar DJ, Frazier OH (2009) LVAD therapy in advanced heart failure. N Engl J Med 361:2241–2251. 10.1056/NEJMoa0909938 19920051

[38] Thuault S, Valcourt U, Petersen M, Manfioletti G, Heldin CH, Moustakas A (2006) TGF-β employs HMGA2 to elicit EMT. J Cell Biol 174:175–183. 10.1083/jcb.200512110 16831886 PMC2064178

[39] Tsao CW, Aday AW, Almarzooq ZI et al (2023) Heart disease and stroke statistics—2023 update. Circulation 147:e93–e621. 10.1161/CIR.0000000000001123 36695182 PMC12135016

[40] Vilahur G, Juan-Babot O, Peña E, Oñate B, Casaní L, Badimon L (2011) Mechanisms of cardiac remodeling after MI. J Mol Cell Cardiol 50:522–533. 10.1016/j.jmcc.2010.12.021 21219908

[41] Wahl SM, Hunt DA, Wakefield LM, McCartney-Francis N, Roberts AB, Sporn MB (1987) TGF-β induces monocyte chemotaxis. Proc Natl Acad Sci U S A 84:5788–5792. 10.1073/pnas.84.16.5788 2886992 PMC298948

[42] Wilson KD, Ameen M, Guo H et al (2020) BANCR promotes cardiomyocyte migration. Dev Cell 54:694-709.e9. 10.1016/j.devcel.2020.07.006 32763147 PMC7529962

[43] Yi X, Li X, Zhou Y, Ren S, Wan W, Feng G, Jiang X (2014) HGF regulates TGF-β1-induced cardiac fibroblast responses. Int J Mol Med 34:381–390. 10.3892/ijmm.2014.1782 24840640 PMC4094591

[44] Zhao W, Wang C, Liu R, Wei C, Duan J, Liu K, Li S, Zou H, Zhao J, Wang L, Qi Y, Liang W, Jiang J, Zhang W, Pang L, Li F (2016) TGF-β1 regulates MSC recruitment after vascular injury. Sci Rep 6:21176. 10.1038/srep21176 26880204 PMC4754777

