# Supplementary material for: TGF-β signalling drives chemotaxis of human induced pluripotent stem cell-derived cardiomyocytes in response to MI stimulus
Source: Basic Res Cardiol. 2026 Aug 12;121(5):1063–76. doi: 10.1007/s00395-026-01201-9 (PMC13593649; doi:10.1007/s00395-026-01201-9)
Supplement: Supplementary file 1 [file 395_2026_1201_MOESM1_ESM.pdf]

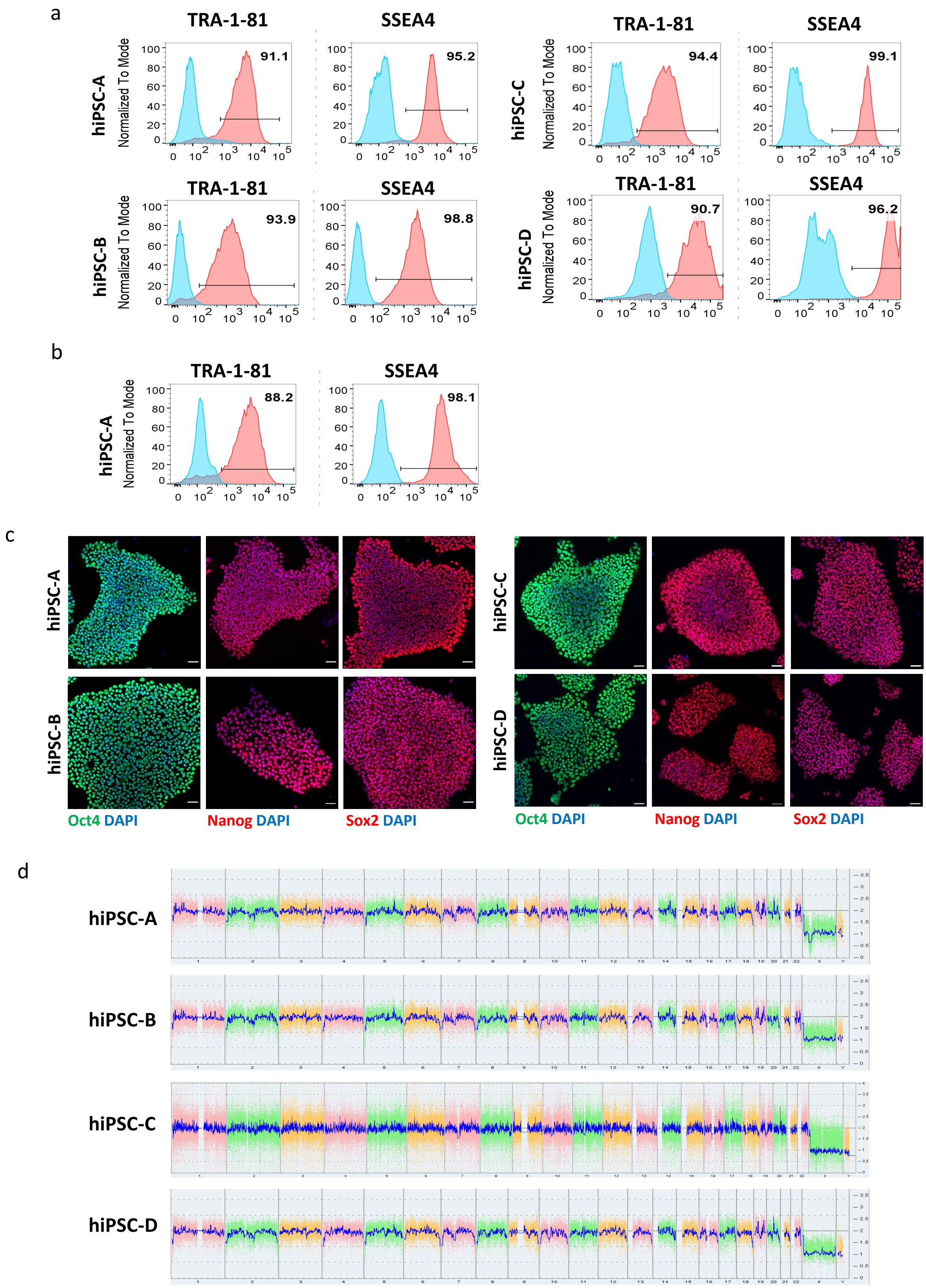

Fig S1 Characterisation of hiPSC lines a) Flow cytometric analysis of pluripotency markers, TRA-1-81 and SSEA4 of hiPSC-A (P47), hiPSC-B (P34), hiPSC-C (P34) and hiPSC-D (P41). Representative histograms show marker expression (red) compared with isotype controls (blue). **b)** Flow cytometric analysis of TRA-1-81 and SSEA4 of hiPSC-A (P77). **c)** Representative micrographs showing immunolabelling of pluripotency markers Oct-4 (green), Nanog (red) and Sox2 (red), nuclei are labelled with DAPI (blue). Scalebar= 50 µm. **d)** hiPSC lines A–D were confirmed to be free of karyotypic abnormalities by KaryoStat analysis at passages 54, 47, 36, and 44, respectively. The whole-genome view displays all autosomal and sex chromosomes in a single plot with corresponding copy number levels. Pink, green, and yellow signals represent raw probe intensities for individual chromosomes, while the blue signal indicates the normalized probe signal used to assess copy number variations and detect deviations from the normal diploid state (CN = 2).

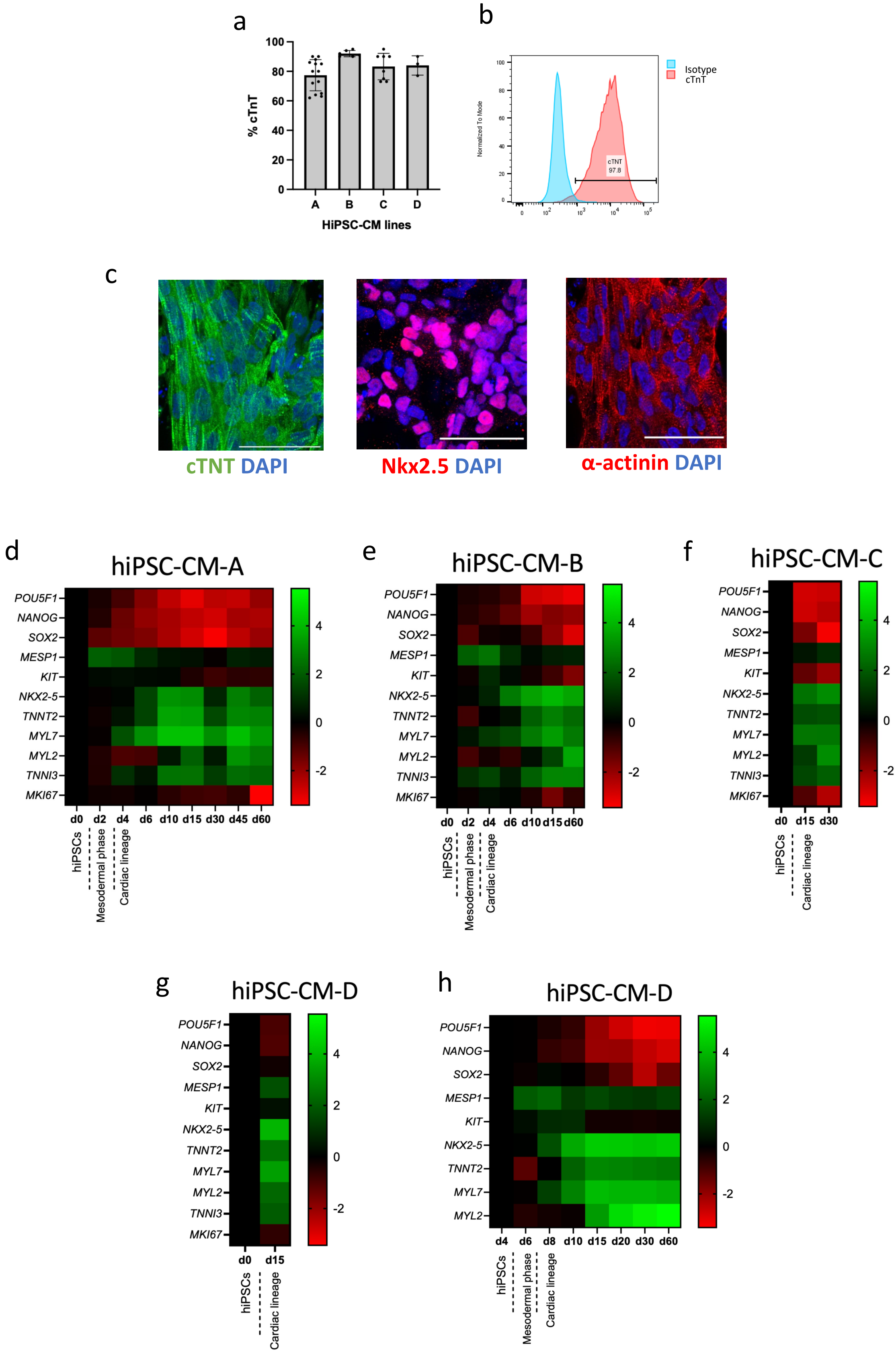

Fig S2 Expression of pluripotency and cardiac markers during hiPSC-CM differentiation A) Differentiation efficiency assessed by cardiac troponin T (cTnT) expression measured by flow cytometry in hiPSC-CM lines A–D generated using the Burridge et al. protocol, bars represent mean ± SD **B)** Representative flow cytometry histogram showing cTnT expression in hiPSC-CM-B at day 15 of differentiation. Samples are shown in red and isotype controls in blue. **C)** Representative micrographs showing immunolabelling of day 15 hiPSC-CM-D using the commercial kit, cTnT (green), Nkx2.5 (red) and α-actinin (red), nuclei are labelled with DAPI (blue). Scalebar= 50 µm. **D-H)** Heat maps illustrating RT-qPCR analysis of pluripotent, mesodermal, and cardiac marker expression during hiPSC-CM differentiation of **D)** hiPSC-CM-A, **E)** hiPSC-CM-B, **F)** hiPSC-CM-C and **G)** hiPSC-CM-D using the Burridge protocol and **H)** hiPSC-CM-D using the commercial kit. Values are represented as log fold change and represent the average of 1-3 samples.

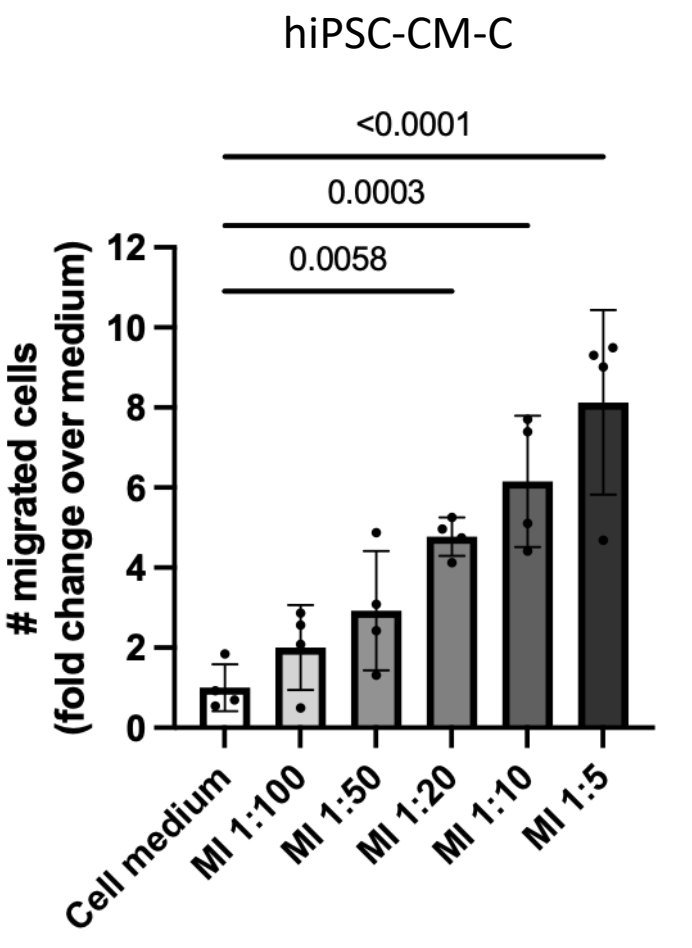

Fig S3 MI homogenate has a concentration-dependent effect on hiPSC-CM-C migration Human iPSC-CM-C were seeded onto transwells, exposed to cell medium alone, non-MI or MI homogenate at varying dilutions for 24h. Non-migrated hiPSC-CMs on the upper surface of the membrane were removed by swabbing and migrated hiPSC-CMs on the lower surface were stained with DAPI and imaged. Bars represent the mean number of nuclei counted across 4 wells per condition. Statistical analysis was performed using one-way ANOVA followed by Dunnett's correction. Error bars indicate mean $\pm$ SD.

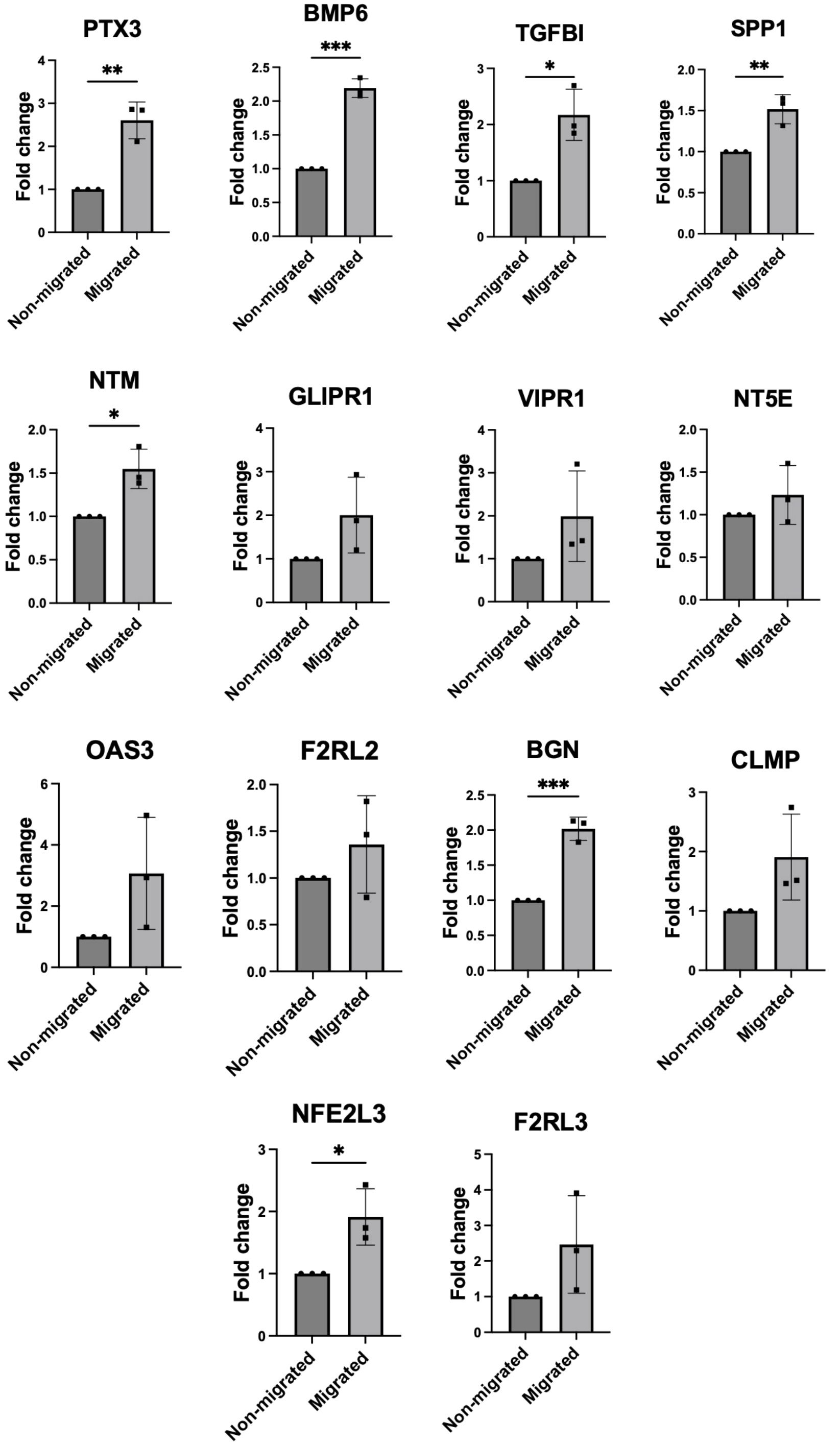

Fig S4 Differentially expressed gene verification comparing migrated hiPSC-CMs to their non-migrated counterparts Human iPSC-CM-A,-B and -C were seeded onto transwells at day 15 and exposed to MI 1:10 for 24h after which the non-migrated and migrated cells were harvested separately. RT-qPCR confirmed the upregulation of all 14 genes of interest in migrated cells compared to their non-migrated counterparts, N=3. Statistical analyses were performed using two-tailed, unpaired t-tests. Error bars indicate mean ± SD. *: P ≤ 0.05, **: P ≤ 0.01, ***: P≤ 0.001.

Table S1 Gene expression profiles of hiPSC-CMs analysed for bulk RNAseq after 24h of incubation with MI homogenate (1:10) Values represent normalised read counts. Padj = adjusted p-value or false discovery rate (FDR).

Gene	hiPSC-CM-A			hiPSC-CM-B		
	Migrated	Non-migrated	padj	Migrated	Non-migrated	padj
TNNT2	8071	18864	0.07	11685	16025	1
NKX2-5	3044	4933	0.48	3892	4316	1
MYL7	18495	44382	0.06	26798	38134	1
NR2F2	1109	487	0.10	746	540	1
MYL2	60	142	0.14	91	123	1
IRX4	126	254	0.23	291	257	1
TBX18	1193	779	0.56	206	85	0.59
HCN4	1458	2882	0.21	1097	1643	1
TNNI3	231	687	0.01	280	515	1
TNNI1	2620	6508	0.05	3499	5198	1
MYH6	26392	88865	0.003	14032	32149	0.53
MYH7	4489	16524	0.001	2285	4259	1
DDR2	806	403	0.21	599	284	0.84
THY1	368	149	0.07	30	5	0.23
CD31	-	-		-	-	

Table S2 Gene Ontology (GO) analysis of migrated versus non-migrated hiPSC-CM-Bs and hiPSC-CM-Cs after 24h of incubation with MI homogenate

GOID	Description	GeneRatio	pvalue	padj
**hiPSC-CM-A**				
GO:0030198	extracellular matrix organization	86/1624	2.29E-15	1.20E-12
GO:0006935	chemotaxis	100/1624	2.08E-09	2.54E-07
GO:0042330	taxis	100/1624	2.34E-09	2.79E-07
GO:2000147	positive regulation of cell motility	90/1624	2.95E-09	3.45E-07
GO:0040017	positive regulation of locomotion	94/1624	3.75E-09	4.19E-07
GO:0031032	actomyosin structure organization	48/1624	8.57E-09	9.18E-07
GO:0030335	positive regulation of cell migration	86/1624	1.88E-08	1.90E-06
GO:0030048	actin filament-based movement	37/1624	1.93E-08	1.91E-06
GO:0060326	cell chemotaxis	41/1624	5.87E-05	0.002
GO:0050920	regulation of chemotaxis	36/1624	7.37E-05	0.002
GO:0050921	positive regulation of chemotaxis	25/1624	0.0003	0.005
GO:0006929	substrate-dependent cell migration	9/1624	0.001	0.01
GO:0042246	tissue regeneration	13/1624	0.001	0.01
GO:0014812	muscle cell migration	17/1624	0.005	0.04
**hiPSC-CM-B**				
GO:0006935	chemotaxis	25/421	0.0048	0.12
GO:0042330	taxis	25/421	0.0049	0.12
GO:2000147	positive regulation of cell motility	21/421	0.012	0.20
GO:0050921	positive regulation of chemotaxis	8/421	0.013	0.20
GO:0030335	positive regulation of cell migration	20/421	0.017	0.23
GO:0040017	positive regulation of locomotion	21/421	0.024	0.28
GO:0060326	cell chemotaxis	11/421	0.028	0.31

Table S3 Differentially expressed genes (DEGs) shared between migrated (A-M,B-M) versus non-migrated hiPSC-CM-A (A-NONM) and hiPSC-CM-B (B-NONM) after 24h of incubation with MI homogenate diluted 1:10 Values represent as normalised read counts. Padj = adjusted p-value or false discovery rate (FDR).

DEG	A-M	A-NONM	log2FC	padj A	B-M	B-NONM	log2FC	padj B
PTX3	4959.38	312.57	3.99	3E-13	1931.98	157.41	3.62	2E-10
SOX17	955.81	83.22	3.52	1E-09	160.50	23.36	2.77	0.0004
BMP6	1081.94	132.95	3.02	2E-07	161.49	32.50	2.31	0.008
TGFBI	9837.96	1522.27	2.69	2E-06	1024.09	229.51	2.16	0.002
SPP1	3503.99	565.27	2.63	3E-06	95.51	10.16	3.22	0.0004
NTM	936.10	133.96	2.80	2E-06	279.65	61.95	2.17	0.006
GLIPR1	1134.16	175.57	2.69	3E-06	192.02	49.76	1.95	0.034
VIPR1	144.85	13.19	3.44	2E-06	67.94	10.16	2.73	0.011
RGPD6	46.31	318.66	-2.78	1E-05	50.22	193.97	-1.95	0.031
NT5E	126.13	27.40	2.20	0.002	240.27	46.72	2.36	0.002
CASP4	217.77	48.71	2.16	0.001	81.73	13.20	2.62	0.009
OAS3	34.49	5.08	2.73	0.012	38.40	0.00	8.27	0.0002
F2RL2	434.55	113.66	1.93	0.002	151.64	31.48	2.26	0.011
BGN	319.26	77.13	2.05	0.001	93.55	17.27	2.43	0.012
CLMP	773.52	259.80	1.57	0.014	509.09	134.05	1.92	0.016
EPAS1	1503.68	459.73	1.71	0.005	208.76	49.76	2.07	0.016
NFE2L3	205.94	37.55	2.45	0.0002	222.54	53.82	2.05	0.016
KCNJ2	76.86	8.12	3.22	9E-05	55.14	8.13	2.74	0.020
KIF18B	692.72	157.30	2.14	0.0003	331.84	88.35	1.91	0.024
MKI67	1768.74	360.27	2.30	6E-05	986.67	286.38	1.78	0.029
HMGA2	11854.04	3122.68	1.92	0.001	5456.22	1629.94	1.74	0.029
VEPH1	59.12	15.22	1.95	0.029	97.48	21.33	2.19	0.029
F2RL3	79.81	23.34	1.77	0.030	100.44	22.34	2.16	0.030
CDK1	1067.16	276.04	1.95	0.001	606.57	176.70	1.78	0.034
SLC7A11	10478.46	3401.76	1.62	0.008	11840.03	3683.36	1.68	0.039
DACT2	102.48	21.31	2.26	0.002	17.72	0.00	7.16	0.044
NEK2	332.07	87.28	1.93	0.003	156.57	40.62	1.94	0.045
DHRS2	19.71	2.03	3.20	0.035	45.29	194.98	-2.10	0.014

Table S4 List of unconjugated antibodies used in this study All antibodies are reactive against human. ICC: immunohistochemistry, WB: western blot, FC: flow cytometry.

Target	Host species	Source	Application	Working concentration
Oct3/4	Goat	R&D, #AF1759	ICC	1:50
Nanog	Goat	R&D, #AF1997	ICC	1:50
Sox2	Rabbit	Abcam, #Ab97959	ICC	1:500
Nkx2.5	Goat	R&D, #AF2444	ICC	1:20
cTNT	Mouse	Thermofisher, #MA5-12960	ICC, WB, FC	1:50
Sarcomeric alpha-actinin	Mouse	Sigma (Merck), #A7811	ICC	1:250
MLC2v	Rabbit	Proteintech, #10906-1-AP	FC	1:400
Phospho-Smad2/3	Rabbit	Thermofisher, #PA5-110155	ICC	1:200

Table S5 List of conjugated antibodies used in this study ICC: immuno-histochemistry, WB: western blot, FC: flow cytometry.

Target	Conjugate	Source	Application	Working concentration
Anti-mouse	AF488	Thermofisher, #A21202	ICC	1:500
Anti-mouse	AF647	Thermofisher, #A21242	FC	1:500
Anti-goat	AF488	Thermofisher, #A11055	ICC	1:500
Anti-goat	AF594	Thermofisher, #A11058	ICC	1:500
Anti-rabbit	AF488	Thermofisher, #A21206	ICC	1:500
Anti-rabbit	AF555	Thermofisher, #A32732	FC	1:500
Anti-rabbit	AF594	Thermofisher, #A11037	ICC	1:500
Anti-human TRA-1-81 (IgM, κ)	AF488	Biolegend, #330710	FC	1:10
Anti-human isotype (IgM, κ)	AF488	Biolegend, #401617	FC	1:10
Anti-human SSEA4 (IgG3)	AF488	Thermofisher, #53-8843-42	FC	1:10
Anti-human isotype (IgG3)	AF488	Biolegend, #401324	FC	1:10
Anti-human cTnT (IgG1)	FITC	Miltenyi, #130-119-575	FC	1:50
Anti-human isotype control (IgG1)	FITC	Miltenyi, #130-118-354	FC	1:50

Table S6 Forward and reverse primer sequences for the genes of interest for RT-qPCR All primers have a melting temperature of approximately 60°C.

	**Primer sequence**	
**Gene**	**Forward (5'-3')**	**Reverse (5'-3')**
POU5F1	CAAAGCAGAAACCCTCGTGC	CTCGGACCACATCCTTCTCG
SOX2	GGGGAAAGTAGTTTGCTGCC	CGCCGCCGATGATTGTTATT
NANOG	CTGCAGAGAAGAGTGTCGCA	ACCAGGTCTTCACCTGTTTGT
MESP1	ACGGTCCCCGCTCCTTC	CAGTCTGCCAAGGAACCACT
KIT	AGGAAACGCTCGACTACCTG	CATTCCAGGATAGGGGCTGC
NKX2-5	CAAGTGTGCGTCTGCCTTTC	CGCACAGCTCTTTCTTTTCGG
TNNT2	ATCCTCTGCCTCACCCAGTC	GCTCTCCCTCAGAACAGCAG
MYL2	CAATTAACTTTACTGTGTTCCTCACAA	GAACGTAATCAGCCTTCAGCAC
MYL7	CCGTCTTCCTCACGCTCTT	TGAACTCATCCTTGTTCACCAC
PTX3	CGTCTCTCCAGCAATGCATC	AAGAGCTTGTCCCATTCCGA
BMP6	CCGACAACAGAGTCGTAATCGC	CTGCCATCCCAGGTCTTGGAAA
TGFBI	ATGACCCTCACCTCTATGTACC	CACAGTTCACAGTTACAATCCCA
SPP1	GCCGAGGTGATAGTGTGGTT	AACGGGGATGGCCTTGTATG
NTM	TGCTGTTCCTTGTACCCACAGG	CGAGGATCCAGGCACCACT
GLIPR1	GGCTTTGACGCTCTTTCCAA	CGTTTGACTTGGTCTCGCTGT
VIPR1	GGACACCATCAACTCCTCACTG	CGCAGTTTCTGAAGCAGGATTCG
NT5E	TCTTCTAAACAGCAGCATTCC	CATTTCATCCGTGTGTCTCAG
OAS3	CCTGATTCTGCTGGTGAAGCAC	TCCCAGGCAAAGATGGTGAGGA
F2RL2	TCCTGGTGTTTGTAGTTGGTGT	CCAGTTGTTCCCATTGAGATGA
BGN	TGACTGGCATCCCCAAAGAC	GAGTAGCGAAGCAGGTCCTC
CLMP	TTACTGGCAGCGAATCCGAGAG	CTGGTACAGTCCAGAGTAGGAC
NFE2L3	CCAGTTGCTTTCATCACAGCCTG	CACATCCTGACTTATAGCCTGGC
F2RL3	ACCGGAGGTGGTGATGACAG	CACAGACTTGGCCTGGGTAG
GAPDH	GTCAAGGCTGAGAACGGGAA	AAATGAGCCCCAGCCTTCTC
ACTB	GTGGCATCCACGAAACTACC	GTACTTGCGCTCAGGAGGAG
